# Numerical Modelling of Geopolymer Concrete In-Filled Fibre-Reinforced Polymer Composite Columns Subjected to Axial Compression Loading

**DOI:** 10.3390/ma15093390

**Published:** 2022-05-09

**Authors:** Varunkumar Veerapandian, Gajalakshmi Pandulu, Revathy Jayaseelan, Veerappan Sathish Kumar, Gunasekaran Murali, Nikolai Ivanovich Vatin

**Affiliations:** 1 Department of Civil Engineering, B.S. Abdur Rahman Crescent Institute of Science & Technology, Chennai 600048, Tamil Nadu, India; varunpandian99@gmail.com (V.V.); revathyj@crescent.education (R.J.); 2 Faculty of Civil Engineering, Architecture and Geodesy, University of Split, 21000 Split, Croatia; 3 Peter the Great St. Petersburg Polytechnic University, 195251 St. Petersburg, Russia; murali_22984@yahoo.com (G.M.); vatin@mail.ru (N.I.V.)

**Keywords:** fibre-reinforced polymer, geopolymer concrete, finite element modelling, fibre orientation, D/t ratio, axial stress distribution, confining pressure

## Abstract

In this research study, the performance of geopolymer concrete (GPC) in-filled fibre-reinforced polymer (FRP) composite (GPC-FRP) columns exposed to compressive loading is examined using the finite element (FE) analysis. The load–deflection behaviour is investigated by considering the impact of the strength of concrete, different fibre orientations and thicknesses of FRP tubes in terms of the diameter/thickness (D/t) ratio, surface friction in between the concrete and enclosing FRP tube, the lateral confinement and the axial stress distribution characteristics. The load-carrying capacity (LCC) of the GPC-FRP composite columns and cement concrete (CC) in-filled FRP composite (CC-FRP) columns is compared and the results imply that the LCC of the GPC-FRP composite columns is (0.9 to 2.04%) greater than the CC-FRP composite columns. The improvement in the LCC and lateral confining pressure of the GPC-FRP composite columns is observed as the thickness of the FRP tube increases. The LCC of the GPC-FRP composite columns with a D/t ratio of 30 was almost (12.70 to 14.23%) greater than the GPC-FRP composite columns with a D/t ratio of 50. The GPC-FRP composite columns with a fibre orientation in the axial and hoop directions (0°) exhibit (8.4 to 11.39%) better performance than the columns with any other orientations (30° and 53°). The LCC of the GPC-FRP composite columns with a coefficient of friction of 0.25 and 0.5 are quite comparable. The axial stress distribution in the GPC-FRP composite columns with different tube thicknesses is explored in this research. This FE model is validated with the experimental results obtained by Kim et al., (2015) and the load and deflection are predicted with the validation error of 6.5 and 6.1%, respectively.

## 1. Introduction

Concrete-filled steel tubes (CFSTs) have been used efficiently as structural members over centuries. Due to their excellent composite behaviour, CFST columns have shown excellent strength towards axial compression, and found a way to be used extensively as columns in structures [1,2,3]. Despite their excellent strength, they have shown various drawbacks, such as the corrosion of steel material in water or moisture-prone areas, unpredicted buckling of steel tubes, etc. By considering the hybrid characteristics of a composite member, the steel in CFSTs was replaced by fibre-reinforced polymer (FRP) composites by researchers, to avoid the aforementioned drawbacks of steel. FRP composites generally show enhanced physical, mechanical and anticorrosive properties, which have found their way to being used as both strengthening members and new tubular column members extensively. The effect of two types of fibre hybridization, including an intrayarn hybrid and interlayer hybrid on the mechanical properties of carbon/glass fibre-reinforced composite rods, was experimentally investigated by calculating the interface/short beam shear strength, three-point bending strength and tensile strength [4]. Guijun et al. [5] used two types of carbon/glass fibre hybrid plates, which included a random fibre hybrid and core–shell hybrid modes exposed to sustainable bending loading and water immersion, and reported that the long-term life prediction of these hybrid plates revealed that random fibre hybrid plates provided a greater corrosive resistance than the core–shell hybrid plates. The strength and durability properties of confined concrete can be substantially enhanced by the lateral confinement of FRP composite tubes, particularly for seismic-resistant structures [6,7]. The seismic dynamic performance of the reinforced concrete structures strengthened with FRP has been investigated by performing linear and nonlinear static analyses, and it has been reported that the strengthening of RC structures with CFRP sheets increases the seismic loading capacity [8].

In the past, several researchers investigated CC-FRP under axial compression [9,10,11,12]. Zeng et al. [13] studied FRP–concrete–steel double-skin tubular columns (DSTC) with a rib-stiffened high-strength steel and ultra-high-strength concrete subjected to axial compressive loading. The quantity and length of the stiffeners, the FRP tube thickness, the steel tube diameter and the concrete strength were the parameters used in this study. The results revealed that these exhibited ductile behaviour and the stiffeners enhanced the performance of the columns. Under axial loading, the performance of FRP confined rectangular concrete-filled thin-walled steel tubular stub columns using high-strength materials was investigated by Du et al. [14]. The parameters, including the aspect ratio of the section, concrete strength and the layers of FRP, were studied and the results demonstrated that the FRP confinement delayed the buckling of the steel tubes and improved the ductility and overall performance of the columns. Nowadays, under axial compression loading, CC-FRP tubular columns using DSTC or high-strength concrete and steel are being employed. The CC-FRP tubular columns without this high-strength concrete and inner steel tube were very limited. The compressive and tensile stresses of GPC are generally higher than CC, which enhances the behaviour of the GPC-FRP composite columns. The creep and shrinkage of GPC is very low, and along with the FRP confinement, the performance of the composite columns can be improved. The GPC is temperature and chemical resistant, and as a composite column, it is capable of performing in any type of climate and area. However, it is to be noted that cement concrete, even though it shows excellent strength and mechanical properties, has raised concerns about the environmental issues it might cause. The manufacturing process of cement causes adverse effects to the atmosphere, since the manufacturing process of cement involves burning the raw materials at 1400 °C, emitting harmful greenhouse gases into the atmosphere. Thus, eco-friendly geopolymer concrete came into existence, having found its way into being used as a structural material in composite structures. Through a literature review, it was found that studies related to the behaviour of FRP columns with in-filled special concrete are comparatively limited. Few researchers have studied the influence of geopolymer concrete over conventional concrete in FRP tubes. Ozbakkaloglu et al. [15] studied the effects of change in the shape of a cross-section of an FRP tube, and revealed that the circular cross-section performed well in various aspects, such as the LCC, improved resistance to rupture, etc. Lokuge et al. [16] used a pultruded type of GFRP short columns, and studied the LCC under axial compression. Ahmed et al. [17] used GFRP bars instead of conventional steel reinforcements and studied their performance towards axial compression and lateral loading. Meena et al. [18] studied the mechanical properties of geopolymer concrete with polypropylene fibres and reported that the mechanical properties and bond strength became enhanced when different types of geopolymer concrete were used by themselves or blended with polypropylene fibres. Trabacchin et al. [19] used basalt FRP bars in geopolymer concrete to investigate the bond behaviour by pull-out tests, relating to the bond strength, bond-slip response and failure mechanisms.

With the advancements in computing systems and application software, the development of numerical modelling applications towards the study of the behaviour of structural elements has also been recently performed by researchers. This would certainly help to investigate/anticipate the designed structural members without the use of any complicated experiments. Several researchers have performed numerical studies on CC-FRP to investigate its behaviour in various aspects, such as the effect of loading, effect of different strengths of in-filled concrete, effect of variations in thicknesses of FRP columns, effect of variation in fibre orientation in FRP tubes, effects of interfacial bonding and steel reinforcement ratio, etc., [20,21,22,23,24,25]. A three-dimensional FE oriented progressive damage model to predict the collapse behaviour of single blade-stiffened composite CFRP panels subjected to uniaxial compressive loading, accounting for both intra and interlaminar damage mechanisms with and without debonding imperfections, has been suggested [26]. An experimental and numerical study on the stability and failure analysis of thin-walled composite columns was performed, and the results demonstrated the relation between buckling, postbuckling and failure characteristics [27]. A two-dimensional FE model was formulated to investigate the influence of a pre-existing imperfection on body armour functionality, and it was observed that the changes in the parameters, such as the size, location and pattern of the pre-existing delamination, affected the ballistic protection efficiency of the laminated armour system [28]. Generally, results from numerical studies have shown excellent accuracy in predicting the behaviours of models, in which several literatures also involved an experimental validation to confirm the same.

Researchers performing the numerical modelling of FRP tubular columns have discerned two major criteria as crucial, viz., the thickness of FRP, orientation of fibres in FRP, material properties, fibre volume fraction and the type of fibre, which greatly influence the performance of the models. The fibres can either be oriented towards the direction of hoop stress/axis or inclined with the axis to a certain angle. According to the test findings, the FRP-wrapped concrete cylinder in the hoop or axial direction performed better than the concrete cylinders wrapped in between the hoop and axial directions or any other orientation [29,30]. The lateral confining pressure would be higher in the FRP tube with the fibre orientations in the hoop and axial directions. Similarly, it has been found that the performance of a column can be improved by increasing the FRP tube thickness [31]. It was reported that the external FRP tube thickness has a considerably more substantial behaviour than the thickness of the inner tube in double-skin FRP composite columns [32]. FRP tubes have mainly been used as they possess high mechanical properties, greatly influencing the performance of the FRP tubes. The mechanical properties differ for different fibre materials. Abbood et al. studied the properties of different FRPs and their constituent materials relating to compressive, shear, flexural and tensile strength [33]. The fibre volume fraction is another parameter that accounts for the performance of the FRP tubes. Generally, tensile strength increases with an increase in the fibre volume fraction. The influence of the fibre volume fraction and orientation on the behaviour of rebar-reinforced ultra-high-performance concrete subjected to uniaxial tensile loading was investigated and, it was reported that tensile strength increased with the increase in fibre volume fraction, and the fibres aligned with the load direction recorded the highest strength [34]. The type of fibre is another parameter that affects the strength of FRP tubes, and it relates to the mechanical properties of the FRP tubes. Vincent et al. studied the effect of the type of fibre on the behaviour of high-strength concrete-filled FRP tubes subjected to concentric compression loading, and the results demonstrated that the type of fibre has a significant influence on the behaviour of high-strength concrete-filled FRP tubes under axial compression loading [35].

This research article studies the behaviour of geopolymer concrete in-filled FRP columns through finite element modelling. The research significance here is the behaviour of GPC-GFRP columns under axial loading with crucial parameters taken into consideration, i.e., the effect of the thickness of the FRP tube and the effect of the different orientation of fibres. The effect of those parameters of axially loaded GPC-GFRP columns is investigated upon the LCC, stress–strain behaviour and fracture mechanisms. The need for a more thorough understanding of the impact of geometric features of the fundamental elements of the GPC-FRP composite columns on the strength of the columns is indicated in the review of the literature. Studies on the use of GPC in FRP tubes require greater insight, and research studies on the performance of these GPC-FRP composite columns subjected to axial compressive loading with these parameters have not been explored much. Against these research necessities, the present study provides an investigation of the performance of composite columns to quantify the effect of CC and GPC, different thicknesses and fibre orientations of FRP tubes. This FE modelling approach could contribute to the simulation and determination of the parameters of composite columns without performing any experiments. Such types of analytical studies would help researchers anticipate the behaviour of GPC-GFRP towards the structural application, which would help to reduce the cost and time of those advanced experiments.

## 2. Research Methodology

**Figure 1:**
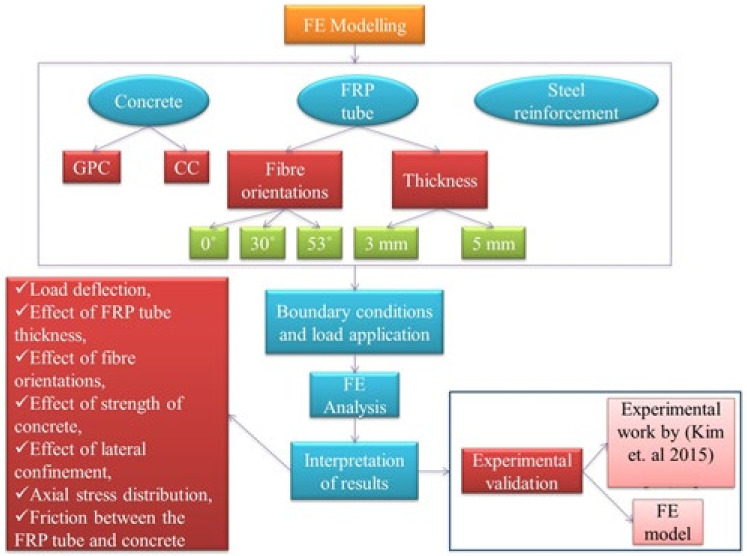
Research methodology [36,37].

Figure 1 shows the detailed research methodology adopted in this paper. The performance of GPC-FRP composite columns under axial compression loading was investigated based on finite element modelling. The behaviour of these GPC-FRP composite columns was examined in terms of loading, deflection and fracture mechanisms. The fibre orientation and thickness of the FRP tubes were chosen by analysing the literature, as well as in regard to the availability. Equal amounts of glass reinforcement were provided for the FRP tubes with each set of thicknesses and fibre orientations. Li et al. [31] studied concrete cylinders wrapped with fibre orientation of 0°, 45° and 90°. To expand the scope of the winding angle findings, three different fibre orientations (0°, 30° and 53°) and two different thicknesses of FRP tubes (3 mm and 5 mm) were considered in this numerical study, in which the behaviour of geopolymer concrete was directly studied in comparison with the behaviour of conventional concrete, being the parameters of FRP material constant. In total, 12 FRP columns were modelled and analysed using ABAQUS software, Dassault Systemes Simulia Corp.: Providence, RI, USA, 2014. (6 GPC-based and 6 CC-based). The circular columns were 1000 mm in length and 150 mm in diameter, respectively. The length and diameter were chosen based on the aspect ratio The aspect ratio (H/D) of the columns was 6.67, and this could be found to be eligible for the composite column to act as a structural member. The 28-day compressive strengths of the GPC and CC used in this numerical modelling were 30.52 MPa and 31.4 MPa, respectively, obtained from experimental trials. Several parameters, such as load–deflection characteristics, the impact of thickness and fibre orientations on the FRP tubes, the axial stress distribution, friction in between the concrete surface and the enclosing FRP tube, and the lateral confinement, were studied. To check the efficiency of these numerical models, experimental studies performed by (Kim et al., 2015) [36,37] were taken into consideration. The parameters used by (Kim et al., 2015) [36,37] were collected from their experimental studies and were incorporated in this numerical modelling, such that the efficiency of those models could be validated with the experimental results from the literature.

## 3. Modelling

### 3.1. Finite Element (FE) Modelling

**Figure 2:**
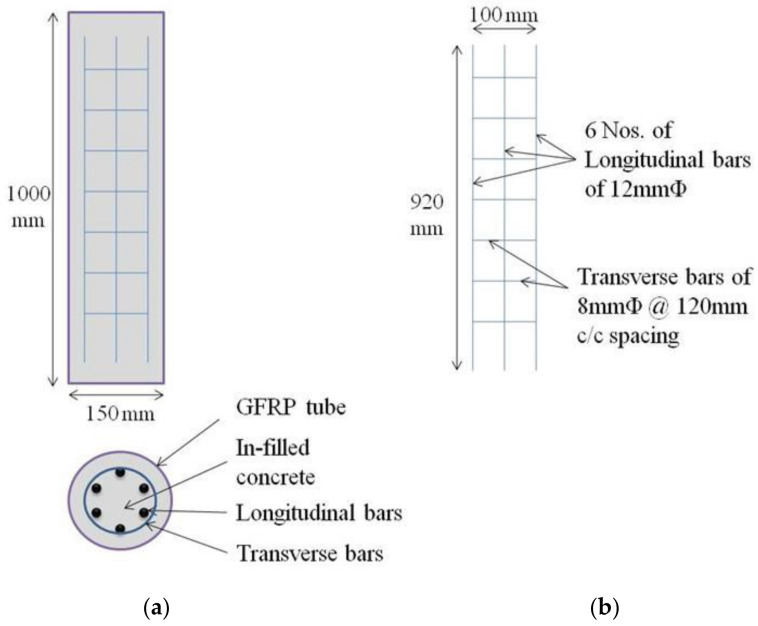

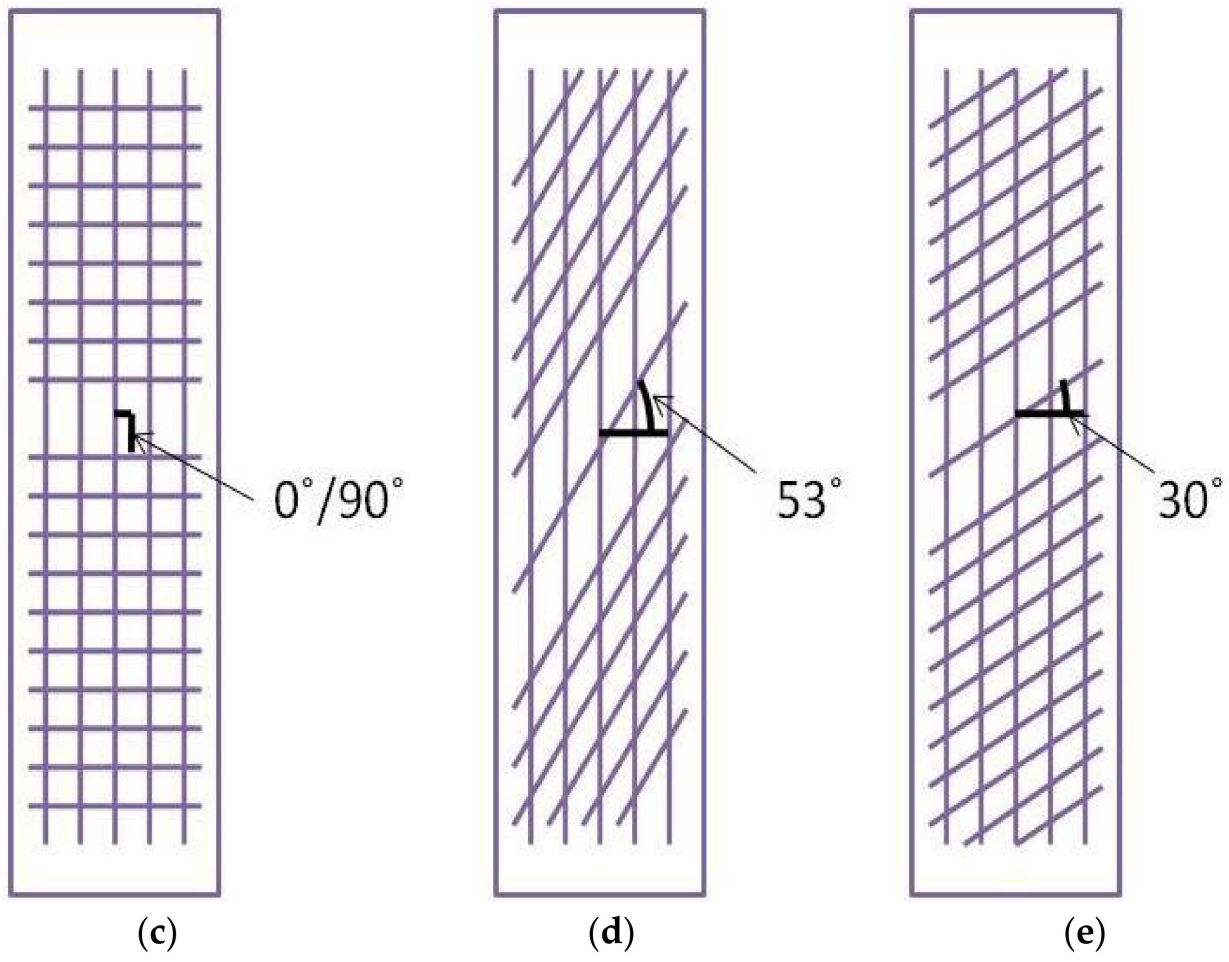
(**a**) Column cross-section, (**b**) reinforcement details and FRP tube with fibre orientation: (**c**) 0°, (**d**) 53° and (**e**) 30°.

The cross-section of the GPC-FRP composite columns, reinforcement details and multiple fibre orientations of FRP tubes are demonstrated in Figure 2. The geometry of the GPC-FRP composite columns with specifications is presented in Table 1.

**Figure 3:**
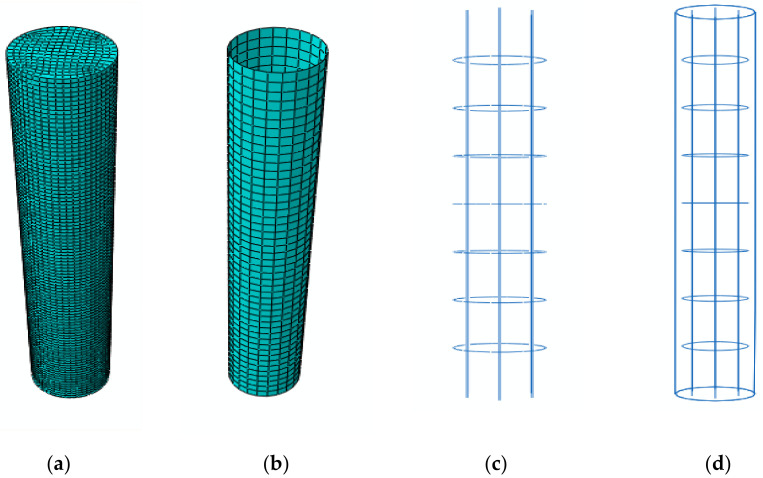
FE components: (**a**) unconfined concrete, (**b**) FRP tube, (**c**) steel reinforcement and (**d**) skeleton section of CC-FRP composite columns.

The test columns were labelled with four characters. The first characters, ‘CC’ or ‘GPC’, denote the cement concrete or geopolymer concrete. The numbers before the letter ‘F’ denote the fibre orientation of the FRP tube (0°, 30° and 53°). The number before the letter ‘T’ denotes the thickness of the FRP tube (3 mm and 5 mm). The FE modelling of the confined concrete, FRP tube, steel reinforcement and the skeleton section of the GPC-FRP composite columns is represented in Figure 3.

### 3.2. Material Modelling

#### 3.2.1. Unconfined Concrete

**Figure 4:**
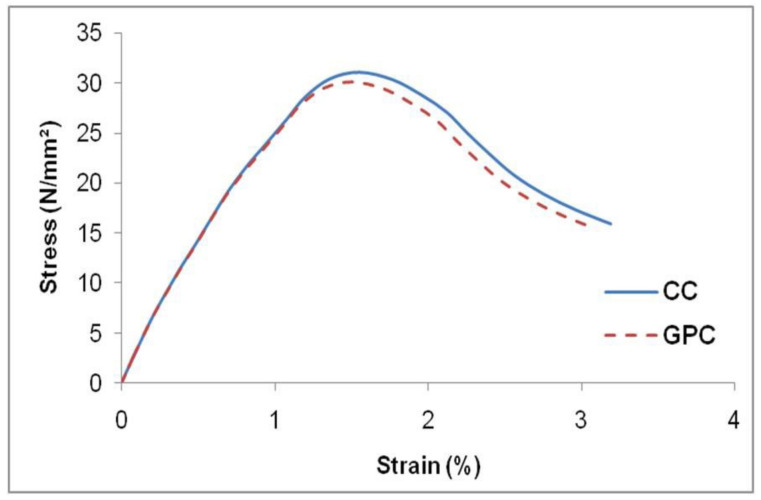
Compressive stress–strain curve of GPC and CC cylinder specimen.

A three-dimensional deformable eight-nodded solid brick element applied with reduced integration was assigned to the model confined concrete [38]. The material properties of the GPC were extracted from the experimental work and are presented in Table 2. The concrete damaged plasticity (CDP) approach [39] was employed to define the plastic property of unconfined GPC. It included a three-dimensional scope of confinement and damage processes, as well as elastic and plastic characteristics. The dilation angle and flow potential eccentricity of the GPC were defined in the CDP parameters. The performance of the FRP-confined GPC could not be replicated by using the elastic properties of unconfined GPC. The GPC stress–strain values were included in the concrete damaged plasticity parameter, and the compression damage was also specified to predict the performance of the confined GPC in GPC-FRP composite columns. A solid homogeneous section was assigned for the section property of GPC-FRP composite columns. The stress–strain curvature was plotted by testing the 28-day compressive strength of the GPC and CC cylinder, as represented in Figure 4.

#### 3.2.2. FRP Tube

An exact categorization of elastic and damage parameters was required to examine the performance of the FRP tubes. To simulate the outer FRP tube, a three-dimensional four-nodded doubly curved shell element with reduced integration was applied. The material type “LAMINA” was used to represent the elastic behaviour of the FRP tubes [40] and the elastic modulus in the hoop and the fibre direction assigned for the FRP tubes. For simulating the elastic characteristics of the FRP tubes, the classical laminate theory [41] was employed. The Hashin damage parameter [42] was adopted in this study to characterize the aspects of damage behaviour in the FRP tubes. This approach figured out the tensile and compressive damage initiation in the fibre and matrix precisely. When additional loading was applied after the damage criterion was initiated, the stiffness coefficients would degrade. These FRP tubes were assigned with the homogeneous shell section and the composite lay-up property. The material characteristics of the FRP tubes are listed in Table 3.

#### 3.2.3. Steel Reinforcement

A three-dimensional truss component with reduced integration was assigned to model the longitudinal and transverse reinforcement bars. The longitudinal reinforcement was provided by a number of six bars of 12 mm diameter and the transverse reinforcement of 8 mm diameter with 120 mm c/c spacing. The elastic material property was assigned to the steel element. The longitudinal and lateral cover was kept at 40 mm and 25 mm, respectively. The tensile strength and the Poisson’s ratio of steel were 411.5 N/mm^2^ and 0.3, respectively.

#### 3.2.4. Surface Interaction, Boundary Conditions and Load Application

The FRP tubes, concrete and the reinforcement steel bars were in contact with each other. The surface interactions between various parts were defined by the interactions manager. The stresses between the different contact surfaces were transmitted by a contact interface. The shear stress between the FRP tube and concrete surface transmitted tangentially. In the tangential direction, the surface contact in between the concrete surface and enclosing FRP tube was described as a frictional interaction with a frictional coefficient of 0.25 and 0.5. To avoid any penetration between the two surfaces, the interaction in the normal direction was categorized as the hard contact. The hard contact would not allow any node to travel from the slave surface to the master surface. The type of contact in between the different surfaces was defined by the master and slave formulation. The master surface was assigned to the concrete surface, whereas the slave surface was assigned to the FRP tube. For defining the contact in between the concrete and reinforcement steel bars, the master surface was assigned to the concrete surface and the slave surface was assigned to the reinforcement bars.

**Figure 5:**
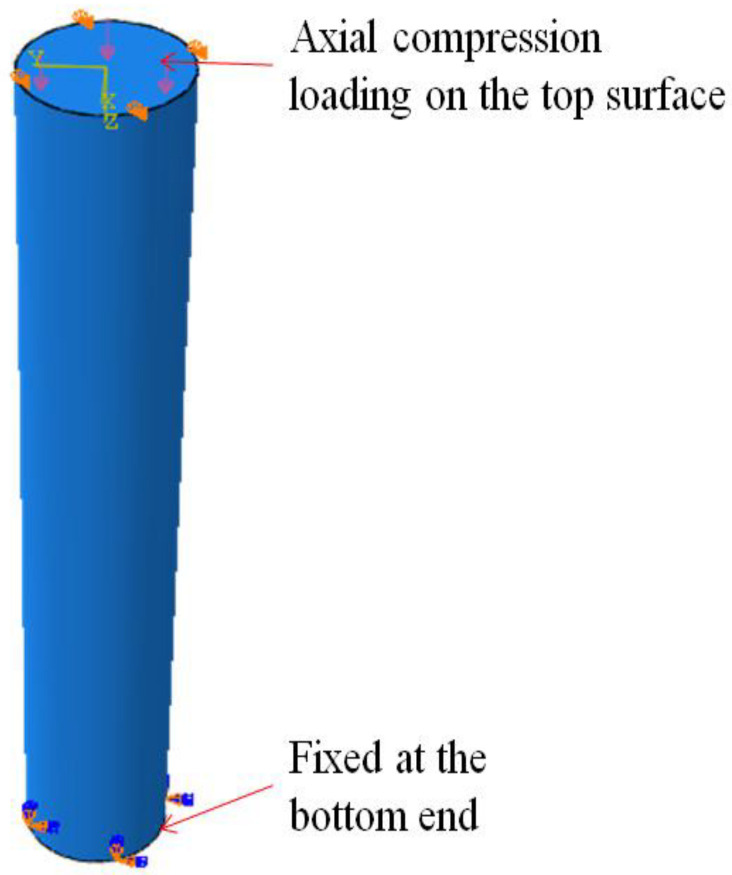
Boundary conditions and load application-defined GPC-FRP composite columns.

Both ends of the column were connected to a point of reference, and the constraints were assigned to the reference points. The column was completely restrained at the bottom end with all the variables and restrained at the top with all the variables except in the longitudinal direction. The displacement loading was imparted on the upper surface as the axial compression. The boundary conditions and load application-defined GPC-FRP composite columns are presented in Figure 5.

## 4. Results and Discussion

### 4.1. Finite Element Modelling Validation

**Figure 6:**
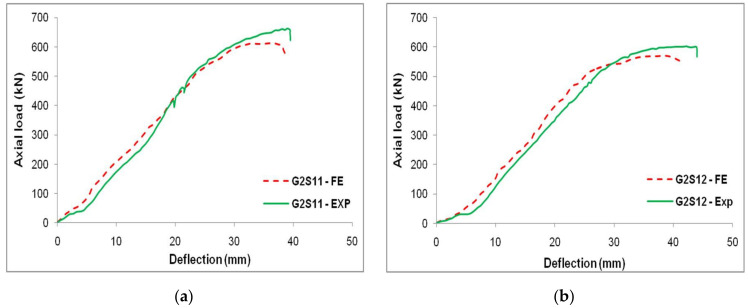
Comparative relation of FE results vs. experimental results obtained by Kim et al. [36,37]; (**a**) G2S11 and (**b**) G2S12.

This FE model was validated by simulating the experimental results reported by Kim et al. [36,37]. From the experiment, two CC-FRP composite columns (G2S11 and G2S12) subjected to compression loading were simulated. The geometry and material properties were extracted from the experimental work by Kim et al. [36,37]. The load–deflection characteristics of these CC-FRP columns subjected to axial loading were studied using experimental data provided by Kim et al. [36,37] and FE findings. The results of these modelled CC-FRP composite columns under axial loading were closely identified along with the experimental results. The average error observed in determining the load and deflection of these CC-FRP columns was 6.5% and 6.1%, respectively. The load–deflection characteristics of the CC-FRP columns of both experimental and FE results are represented in Figure 6.

**Figure 7:**
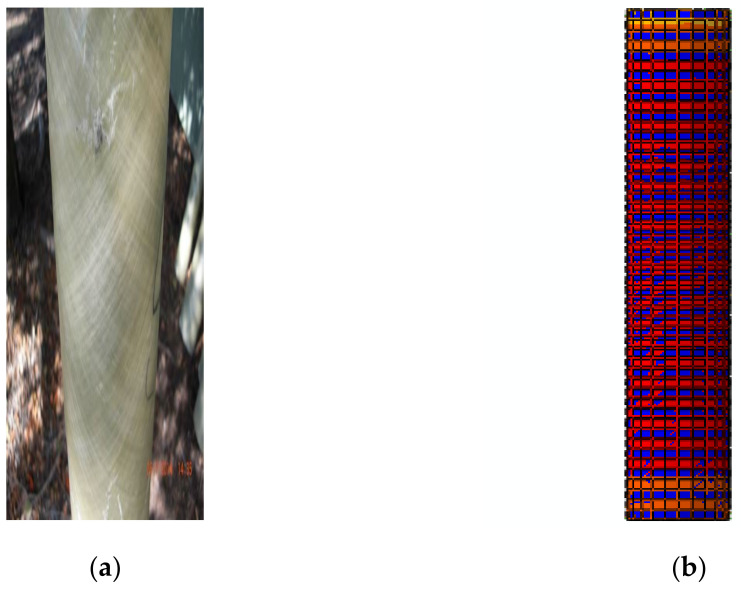
Observed changes in the FRP texture (**a**) experimental work by Kim et al. [36,37] and (**b**) FE model.

**Figure 8:**
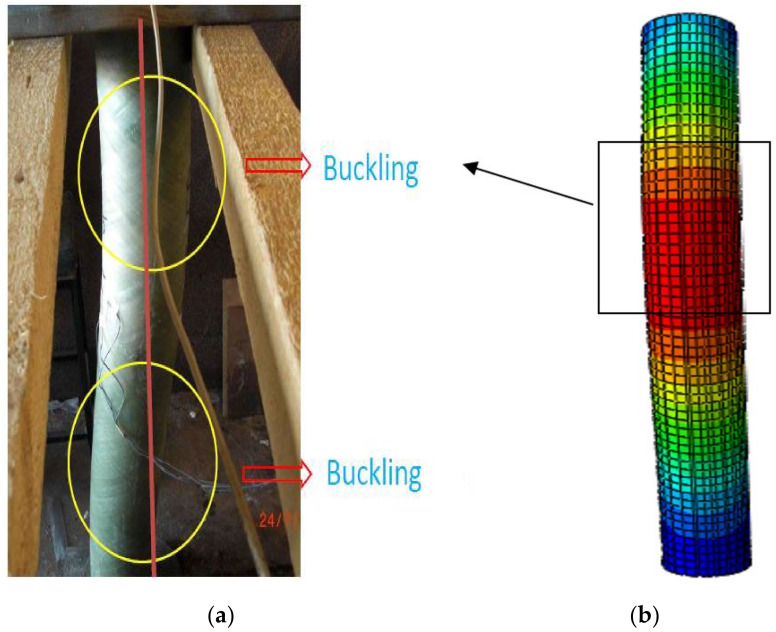
Observed buckling in the CC-FRP columns in (**a**) experimental work by Kim et al. [36,37] and (**b**) FE model.

**Figure 9:**
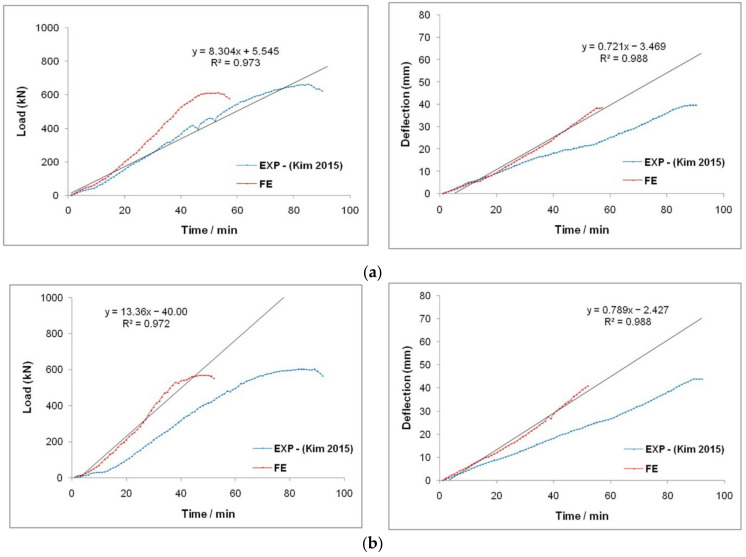
Experimental [36,37] and FE results of the columns (**a**) G2S11 and (**b**) G2S12.

The results indicated that the colour and texture of the FRP tube changed when it was compressed under axial loading, which is presented in Figure 7. From the load–deflection characteristics, the increment in the load exhibited a linear relationship with the displacement, and when it reached the yield point and dropped instantly. The failure of the CC-FRP composite columns occurred mainly due to buckling, as shown in Figure 8. The buckling was noticed at the mid-portion of the FRP tubes in both the FE simulation and the experimental work carried out by Kim et al. [36,37]. The comparison of load–deflection with respect to time of both experimental works by Kim et al. [36,37] and the FE simulation is represented in Figure 9.

### 4.2. Load–Deflection Behaviour

**Figure 10:**
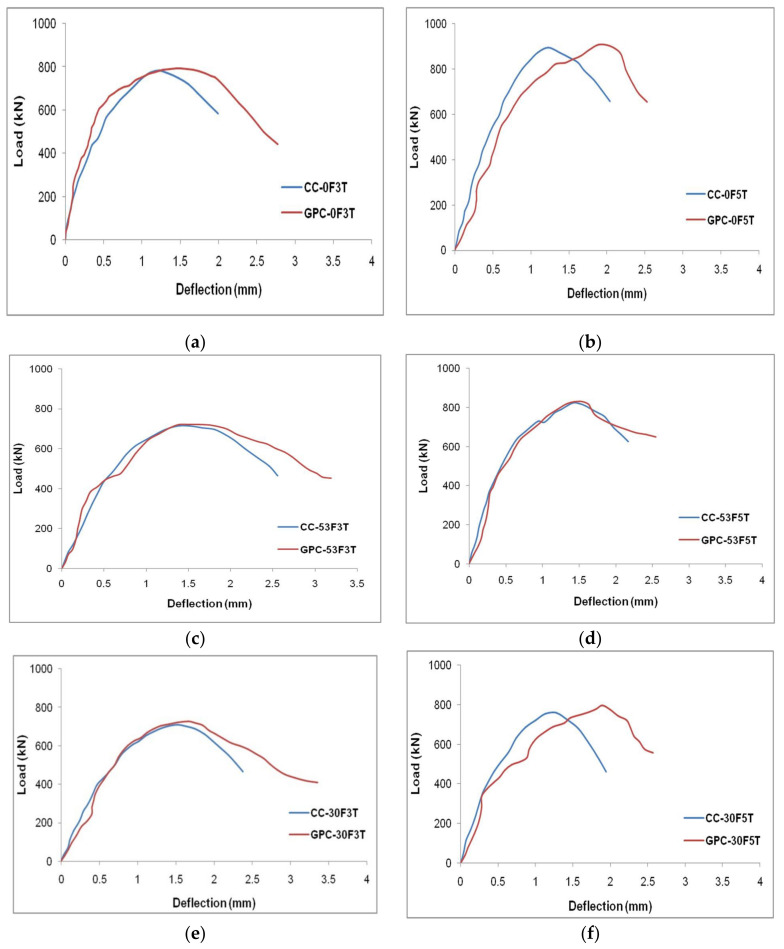
Load–deflection curves of GPC-FRP composite columns; (**a**) CC-0F3T and GPC-0F3T, (**b**) CC-0F5T and GPC-0F5T, (**c**) CC-53F3T and GPC-53F3T, (**d**) CC-53F5T and GPC-53F5T, (**e**) CC-30F3T and GPC-30F3T and (**f**) CC-30F5T and GPC-30F5T.

The load–deflection data of the CC-FRP and GPC-FRP composite columns with various thicknesses and fibre orientations are represented in Figure 10. The load and deflection of the CC-FRP and GPC-FRP composite columns had a linear correlation in the initial loading procedure. After yielding, the load increased linearly, resulting in the second linear component of the curve. Following that, the load reached its maximum capacity as an outcome of the fracture of the FRP tubes, which caused the load to drop immediately, as seen in Figure 10. The specimen C-0F5T exhibited the highest LCC (907.39 kN) of all the GPC-FRP composite columns. The LCC and concrete confinement enhanced as the thickness of the FRP tube increased. The LCC of the GPC-FRP composite columns with a fibre orientation of 0° exhibited greater ductility than the specimens with fibre orientations of 53° and 30°. Specimen CC-30F3T exhibited the lowest load-carrying capacity (714.69 kN) out of all the other column specimens.

In Figure 10a, the load–deflection curve of column GPC-0F3T is presented, which shows a linear elastic part of increasing the axial load, and the curve attained the yield point. This linear part indicated the stiffness of the composite columns under axial load. The load increased after reaching the yield point and dropped gradually after reaching the peak axial load. The load drop was mainly due to the failure of the column subjected to the rupture of the FRP tube. The deflection increased when the load reached the peak point. The GPC-FRP composite columns with a thickness of 3 mm possessed similar load–deflection characteristics.

The load–deflection curve of column GPC-0F5T is shown in Figure 10b. There was a greater ascending elastic part where the load increased gradually and reached the yield point. The increase in the elastic part was mainly due to the increase in the thickness of the FRP tube. The axial load further reached the peak load and dropped immediately. This load drop was due to the rupture of the FRP tube caused by the in-filled concrete compression. This load–deflection behaviour of the GPC-FRP column with a thickness of 5 mm exhibited similar characteristics to column GPC-0F5T.

### 4.3. Effect of Strength of GPC and CC

Under axial loading, the performance of the GPC-FRP and CC-FRP composite columns was compared and tabulated in Table 4. It can be seen that the ultimate LCC of the GPC-FRP composite columns was higher than the CC-FRP composite columns. The LCC of column GPC-0F3T was 0.91% higher than column CC-0F3T. Column GPC-0F5T exhibited a 1.01% higher LCC than column CC-0F5T. The maximum LCC of column GPC-53F3T was 1.42% higher than column CC-53F3T. Column GPC-53F5T exhibited a 2.04% higher LCC than column CC-53F5T. The maximum LCC of column GPC-30F3T was 0.90% higher than column CC-30F3T. Column GPC-30F5T exhibited a 1.11% higher LCC than column CC-30F5T. The results demonstrated that the GPC-FRP composite columns had a slightly greater ultimate LCC than the CC-FRP composite columns.

The GPC-FRP composite columns and CC-FRP composite columns exhibited a similar failure pattern. The crushing of the in-filled concrete was caused by the axial compression, followed by the rupture of the FRP tubes confining this in-filled concrete. The multiple ruptures in the FRP tubes observed in the GPC and CC-FRP columns with a D/t ratio of 50 were caused by the excessive internal stresses. The failure at the ends would be greater than at the centre of the column due to the effective confinement and bonding at the centre.

### 4.4. Impact of D/t Ratio

**Figure 11:**
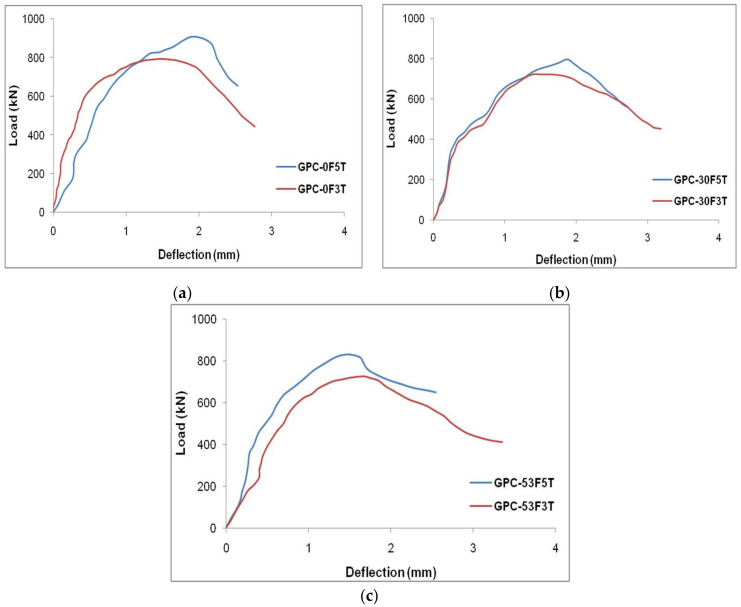
Impact of tube thickness of GPC-FRP composite columns with fibre orientation: (**a**) 0°, (**b**) 30° and (**c**) 53°.

The impact of the D/t ratio on the GPC-FRP composite column related to the LCC and associated deflection was studied. The GPC-FRP composite columns with a D/t ratio of 30 (GPC-0F5T, GPC-30F5T and GPC-53F5T) had a better LCC than the GPC-FRP composite columns with a D/t ratio of 50 (GPC-0F3T, GPC-30F3T and GPC-53F3T). The maximum LCC of column GPC-0F5T was 12.70% greater than that of column GPC-0F3T. The maximum LCC of column GPC-30F5T was 10.25% higher than that of column GPC-30F3T. Column GPC-53F5T exhibited a 14.23% higher LCC than column GPC-53F3T. In comparison to the column with a D/t ratio of 50, the GPC-FRP composite column with a D/t ratio of 30 had a greater load LCC and lesser deflection. When compared to other columns, the FRP tubes with a lower thickness exhibited greater ductility. The impact of the thickness of the FRP tubes on the load–deflection characteristics of the GPC-FRP composite columns with different fibre orientations (0°, 30° and 53°) is represented in Figure 11.

The impact of the D/t ratio was purely related to the tensile strength of the FRP tube. The GPC-FRP composite column with a greater tensile strength enhanced the performance of the columns. The tensile strength of the FRP tube with a D/t ratio of 30 was greater than that of the FRP tube with a D/t ratio of 50. Similarly, the performance of the GPC-FRP composite column with a D/t ratio of 30 was greater than the columns with a D/t ratio of 50.

### 4.5. Axial Stress Distribution

**Figure 12:**
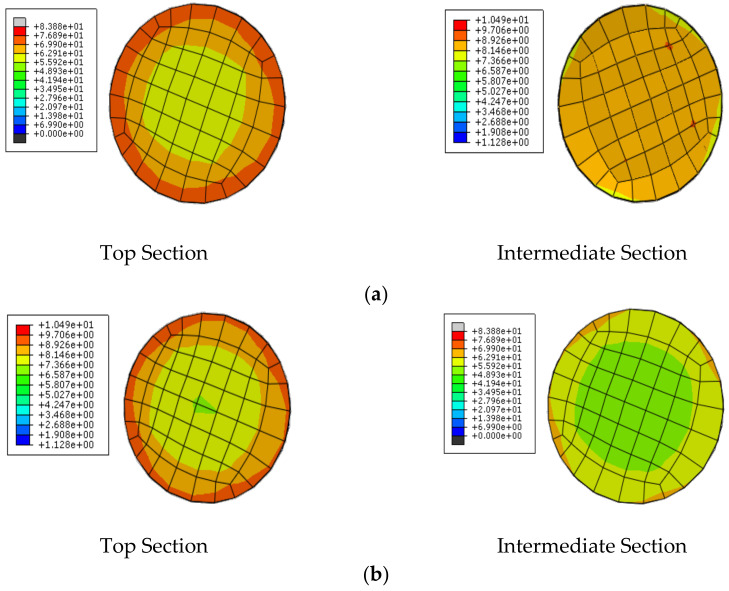
Axial stress distribution in the top and intermediate sections of the GPC-FRP composite columns with tube thicknesses (**a**) GPC-0F3T and (**b**) GPC-OF5T.

As the GPC-FRP composite columns were subjected to axial compression loading, the axial stress distribution could demonstrate the compressive stress caused due to the application of compression loading at different points. The distribution of axial stress in the top and middle cross-sections of GPC-FRP composite columns GPC-0F3T and GPC-0F5T is presented in Figure 12. The stress distribution pattern of the GPC-FRP columns GPC-53F3T and GPC-30F3T was similar to column GPC-0F3T. Columns GPC-53F5T and GPC-30F5T exhibited identical stress distribution patterns to column GPC-0F5T. The stress distribution zone in the GPC-FRP composite columns decreased when the FRP tube thickness increased. It was observed from Figure 11a that the intensity of stress gradually decreased from the edge towards the centre. The main cause for the variation in the stress distribution between the top and middle surfaces was identified to be the poor confinement of FRP tubes with the in-filled concrete on the surface, which was highly efficient in the middle. Due to this peculiar behaviour of the confinement of FRP tubes with in-filled concrete, the GPC-GFRP columns showed excellent resistance towards fractures and buckling.

### 4.6. Impact of Fibre Orientation

**Figure 13:**
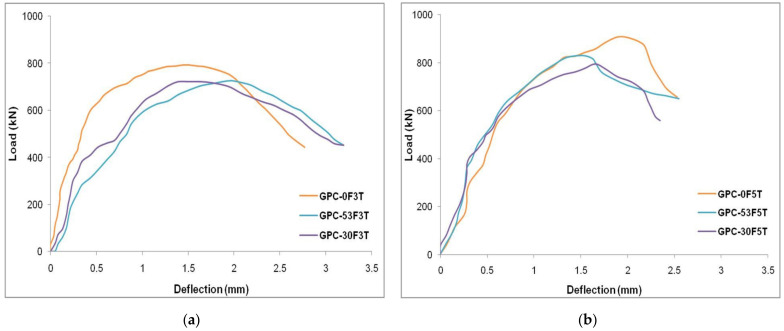
Effect of fibre orientation of column specimen with (**a**) 3 mm thickness and (**b**) 5 mm thickness.

The different fibre orientations (0°, 30° and 53°) of the GPC enclosing the FRP tubes influenced the performance of the GPC-FRP composite columns significantly. It was crucial to analyse the effect of fibre orientations on the behaviour of GPC-FRP composite columns. Among the GPC-FRP composite columns of 3 mm thickness, the maximum LCC of column GPC-0F3T was 9.84% greater than column GPC-30F3T and 8.94% higher than column GPC-53F3T. Among the GPC-FRP composite columns of 5 mm thickness, the LCC of column GPC-0F5T was 14.13% greater than column GPC-30F5T and 9.25% greater than column GPC-53F5T. The fibre orientation in the hoop direction (0°) appeared to contribute to the increased ultimate load. The impact of different fibre orientations (0°, 30° and 53°) related to the load–deflection characteristics of the GPC-FRP composite columns with different thicknesses (3 mm and 5 mm) is represented in Figure 13.

The FRP tube with the fibre orientation of 0° possessed greater tensile strength than the fibre orientations of 30° and 50°. This was mainly due to the alignment of fibres in the axial or hoop direction, which better enhanced the confinement than any other fibre alignments. As the compressive strength of concrete and the grade of steel was similar for the GPC-FRP composite columns, the performance depended on the material properties of the FRP tubes. The FRP tube with better material properties contributed to the better performance of GPC-FRP composite columns.

### 4.7. Friction in between the Concrete Surface and Enclosing FRP Tubes

A frictional coefficient was required in the FE modelling to simulate the interfacial behaviour with the help of Coulomb’s theory of friction. There was an interfacial contact in between the surface of the concrete and FRP tube. This contact could be defined in the form of a coefficient of friction in order to assess the interfacial behaviour and failures. The performance of the GPC-FRP composite columns with different thicknesses (3 mm and 5 mm) and fibre orientations (0°, 30° and 53°) was evaluated by varying the coefficient of friction in between the concrete surface and enclosing FRP tubes. The coefficient of friction in between the concrete surface and enclosing FRP tubes was set at 0.25 and 0.5. The load–deflection behaviour did not make a difference with the frictional coefficient of 0.25 and 0.5. It could be concluded that these changes in the surface interactions had very little impact on the LCC and corresponding deflection of GPC-FRP composite columns.

### 4.8. Impact of FRP Confinement

**Figure 14:**
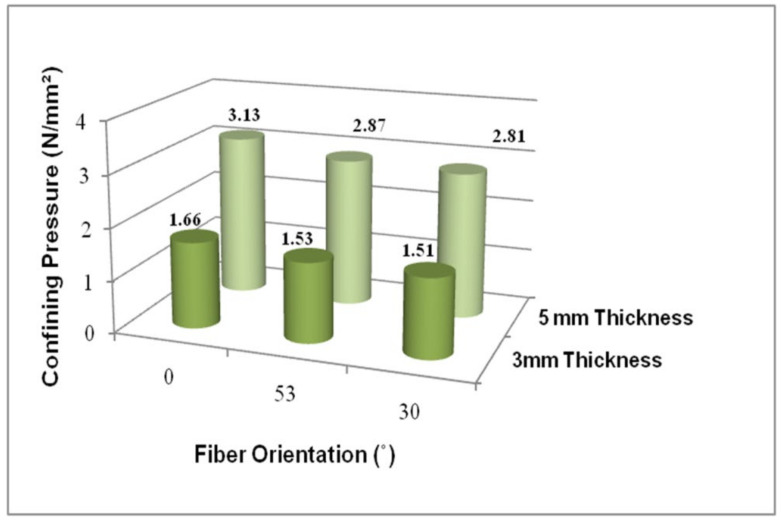
Lateral confining pressure of GPC-FRP composite columns with different fibre orientations (0°, 53° and 30°) and different thicknesses (5 mm and 3 mm).

In the GPC-FRP composite columns, the confined concrete tended to expand, and this was restricted by the FRP tubes when subjected to axial loading. This lateral confining was given by:

(1) $$f_{l}=\frac{2t_{frp}f_{frp}}{d}$$

where $f_{l}$ denotes the lateral confining pressure, $t_{frp}$ and $f_{frp}$- are the thickness and the tensile strength of the FRP tubes and d denotes the diameter of the concrete, respectively [43]. The lateral confining pressure of the GPC-FRP composite columns with different thicknesses (3 mm and 5 mm) and fibre orientations (0°, 30° and 53°) is represented in Figure 14.

The lateral confining pressure of the GPC-FRP composite column GPC-0F5T was 88.6% higher than column GPC-0F5T. Column GPC-53F5T exhibited an 87.6% higher lateral confining pressure than column GPC-53F3T. Column GPC-30F5T exhibited an 86.1% higher lateral confining pressure than column GPC-30F3T. The lateral confining pressure of the GPC-FRP composite columns improved with an increase in the FRP tube thickness. However, this lateral confinement was proportional to the tensile strength of the FRP tube.

## 5. Conclusions

Under axial compression loading, the performance of the GPC-FRP composite columns was investigated. To carry out the FE simulation on the behaviour of these GPC-FRP composite columns, ABAQUS software was used. A total of twelve GPC-FRP composite columns was examined to evaluate the crucial aspects that may have an impact on the behaviour of the GPC-FRP composite columns under compression loading. The strength of the GPC and CC, the effect of the orientation of fibres in FRP tubes, the layer thickness of the FRP tube and the axial stress distribution and the friction in between the concrete surface and the FRP tube on the behaviour of the GPC-FRP composite columns were investigated.

- The LCC of the GPC-FRP composite columns (GPC-0F3T, GPC-0F5T, GPC-53F3T, GPC-53F5T, GPC-30F3T and GPC-30F5T) was (0.91, 1.01, 1.42, 2.04, 0.90 and 1.11%) greater than the CC-FRP composite columns (CC-0F3T, CC-0F5T, CC-53F3T, CC-53F5T, CC-30F3T and CC-30F5T).
- The ultimate LCC and corresponding deflection of the GPC-FRP composite columns could be considerably enhanced by increasing the thickness of the shell.
- The LCC of the GPC-FRP composite columns with a D/t ratio of 30 (GPC-0F5T, GPC-30F5T and GPC-53F5T) was (12.70, 10.25 and 14.23%) greater than the GPC-FRP composite columns with a D/t ratio of 50 (GPC-0F3T, GPC-30F3T and GPC-53F3T).
- The GPC-FRP composite columns with a fibre orientation of 0° exhibited better performance than the GPC-FRP composite columns with other fibre orientations (30° and 53°).
- Among the GPC-FRP composite columns with a D/t ratio of 50, the maximum LCC of column GPC-0F3T was (9.84 and 8.94%) greater than the columns GPC-30F3T and GPC-53F3T.
- In the GPC-FRP composite columns with a D/t ratio of 30, column GPC-0F5T was (14.13 and 9.25%) greater than columns GPC-30F5T and GPC-53F5T.
- The axial stress distribution pattern was higher in the GPC-FRP composite columns with 3 mm thickness than the GPC-FRP composite columns with 5 mm thickness.
- The load-carrying efficiency of the GPC-FRP composite columns with friction coefficients of 0.25 and 0.5 was quite comparable.
- The lateral confining pressure of the GPC-FRP composite columns improved as the thickness of the FRP tubes increased, and it was closely dependant on the tensile characteristics of the FRP tube.
- In the GPC-FRP composite columns with a D/t ratio of 50, the lateral confining pressure of column GPC-0F3T was (8.4 and 8.6%) higher than columns GPC-53F3T and GPC-30F3T.
- Among the GPC-FRP composite columns with a D/t ratio of 30, column GPC-0F5T was (9.06 and 11.39%) greater than columns GPC-53F5T and GPC-30F5T.
- This FE model was verified with the experimental results acquired by Kim et al., (2015) [36,37], and the FE results were comparable and closely related to the experimental work. This FE model employing ABAQUS software could be adapted to replicate the performance of GPC-FRP composite columns.

## Figures and Tables

**Table 1:** Geometry of the column specimens with specifications.

Column Label	Concrete Type	FRP Type	FRP Tube Thickness (mm)	D/t Ratio of FRP Tube
CC-0F3T	CC	GFRP	3	50
CC-0F5T			5	30
CC-53F3T			3	50
CC-53F5T			5	30
CC-30F3T			3	50
CC-30F5T			5	30
GPC-0F3T	GPC		3	50
GPC-0F5T			5	30
GPC-53F3T			3	50
GPC-53F5T			5	30
GPC-30F3T			3	50
GPC-30F5T			5	30

**Table 2:** Material properties of GPC and CC.

Material Properties	Density (kg/m³)	Compressive Strength (MPa)	Elastic Modulus (GPa)	Poisson’s Ratio
GPC	2380	30.52	27.39	0.21
CC	2412	31.40	28.02	0.23

**Table 3:** Material characteristics of FRP tubes.

Type of FRP Material	Thickness (mm)	Fibre Orientation	Tensile Modulus (MPa)	Tensile Strength (MPa)	Elongation (%)
GFRP	3	0°	4158.00 (22.05)	41.44 (1.22)	1.56
		53°	2567.00 (42.87)	38.16 (0.73)	1.87
		30°	2320.83 (41.64)	37.78 (0.53)	0.88
	5	0°	4537.74 (36.74)	46.89 (0.98)	5.55
		53°	4224.78 (26.94)	43.11 (1.10)	7.13
		30°	4205.79 (30.62)	42.10 (0.92)	3.90

The values in the bracket were the standard deviation.

**Table 4:** Test results of GPC-FRP composite columns.

Type of Column	Ultimate Load (kN)	Deflection (mm)	Lateral Confining Pressure	Failure
CC-0F3T	784.99	2.74	1.66	FRP Rupture
CC-0F5T	898.32	2.48	3.13	
CC-53F3T	716.91	3.07	1.53	
CC-53F5T	813.93	2.41	2.87	
CC-30F3T	714.69	3.22	1.51	
CC-30F5T	786.32	2.51	2.81	
GPC-0F3T	792.12	2.77	1.66	
GPC-0F5T	907.39	2.54	3.13	
GPC-53F3T	727.09	3.19	1.53	
GPC-53F5T	830.54	2.52	2.87	
GPC-30F3T	721.18	3.36	1.51	
GPC-30F5T	795.07	2.56	2.81	

## Data Availability

Not applicable.
